# Oncotoxic Properties of Serotonin Transporter Inhibitors and 5-HT_1A_ Receptor Ligands

**DOI:** 10.3390/ijms19103260

**Published:** 2018-10-20

**Authors:** Jarosław Walory, Lidia Mielczarek, Małgorzata Jarończyk, Mirosława Koronkiewicz, Jerzy Kossakowski, Ryszard Bugno, Andrzej J. Bojarski, Zdzisław Chilmonczyk

**Affiliations:** 1 Department of Drug Biotechnology and Bioinformatics, National Medicines Institute, Chełmska 30/34, 00-725 Warsaw, Poland; l.mielczarek@nil.gov.pl (L.M.); mjaronczyk@gmail.com (M.J.); m.koronkiewicz@nil.gov.pl (M.K.); z.chilmonczyk@nil.gov.pl (Z.C.); 2 Warsaw University of Medicine, Banacha 1, 02-097 Warsaw, Poland; jerzy.kossakowski@wum.edu.pl; 3 Department of Medicinal Chemistry, Institute of Pharmacology, Polish Academy of Sciences, Smętna 12, 31-343 Cracow, Poland; bugno@if-pan.krakow.pl (R.B.); bojarski@if-pan.krakow.pl (A.J.B.)

**Keywords:** 5-HT_1A_ receptor, apoptosis, cancer, cytotoxic activity, serotonin ligands, SH-SY5Y, SSRI, S14506, PC-3

## Abstract

The cytotoxic activity of several serotonin transporter (SERT) inhibitors and subtype of serotonin receptor 1A (5-HT_1A_ receptor) ligands have been examined in androgen-insensitive human PC-3 prostate and neuroblastoma SH-SY5Y cancer cells. Almost all of the studied compounds (except 5-HT_1A_ receptor agonist (2*R*)-(+)-8-Hydroxy-2-(di-*n*-propylamino)tetralin hydrobromide (8-OH-DPAT)) exhibited absolute cytotoxic activity against the examined cancer cells. The compound 4-Fluoro-*N*-[2-[4-(7-methoxy-1-naphthalenyl)-1-piperazinyl]ethyl]benzamide hydrochloride (S14506) that showed highest activity against neuroblastoma tumors was the 5-HT_1A_ receptor agonist (although not alike other 5-HT_1A_ receptor agonists). On the other hand, the compound 6-nitro-2-(4-undecylpiperazin-1-yl)quinoline hydrochloride (AZ07) that had the highest activity against PC-3 prostate cancer cells was a compound exhibiting antagonistic activity against the 5-HT_1A_ receptor. Thus, compounds of oncotoxic properties S14506 and AZ07 should be evaluated further for their potential use in the prevention and treatment of cancer. Most of the 15 compounds tested exhibited either agonistic or antagonistic activity for both the cyclic adenosine monophosphate (cAMP) and extracellular signal-regulated kinase 1 and 2 (ERK1/2) pathways in human embryonic kidney 293 (HEK293) cells that overexpress the *5HT1AR* gene. However, compounds paroxetine, *N*-Ac-paroxetine and 2-[4-(cyclobutylmethyl)piperazin-1-yl]-6-nitroquinoline hydrochloride (AB22) simultaneously exhibited antagonistic activity on the cAMP pathway and agonistic activity on the ERK1/2 pathway. Fluoxetine relative to compound AZ07 had almost three times lower cytotoxic activity against PC-3 prostate cancer cells. However, the proapoptotic activity of fluoxetine compared to compound AZ07 is almost two times higher which would suggest that the cytotoxic activity of both compounds may be dependent on different cell death mechanisms. Compound S14506 was found to be an antagonist of the serine-threonine protein kinase B (Akt) pathway. Prosurvival Akt activity may be reversed by Akt antagonists. Therefore, the antagonistic activity of S14506 on the Akt pathway may evoke caspase-3 expression and cytotoxicity. It appears that one should not expect a straightforward relationship between the activation of particular serotonergic pathways by selective serotonin reuptake inhibitors (SSRIs) and 5-HT_1A_ receptor ligands and their cytotoxic or cytoprotective activity. Additionally, nuclear transcription factor κB (NF-κB), which may be involved in 5-HT-dependent biochemical pathways by coordinating different subunits in the formation of a dimer, may regulate the transcription of different transduction pathways. Therefore, it can be suggested that the mechanism of the cytotoxic activity of certain compounds (serotonergic against nonserotonergic) may depend on the compound and cancer type being examined. Docking studies showed that S14506, buspirone and spiperone bind in similar ways in the 5-HT_1A_ receptor model and interacted with similar 5-HT_1A_ receptor residues. S14506 and spiperone were found to be located closer to both phenylalanines in TM6 than buspirone, thus exhibiting more antagonist binding modes.

## 1. Introduction

Selective serotonin reuptake inhibitors (SSRIs) are among the most commonly used antidepressant drugs [1]. It has been shown that some antidepressant drugs and some subtype of serotonin 1A (5-HT_1A_) receptor ligands may exhibit neuroprotective activity, which could be connected to their antidepressant activity. On the other hand, it was reported that the very same group of compounds may induce apoptosis in some cancer cell lines (for a review see [2]). This activity could be connected to the compounds’ serotonergic activity since it was found that 5-hydroxytryptamine (5-HT) induced proliferation and migration of PC-3 and DU-145 prostate cells (but not androgen-dependent LNCaP prostate cells). The action of 5-HT was inhibited to varying degrees by the inhibition of mitogen activated protein kinase (MAPK) and phosphatidylinositol-3-kinase (PI3K) as well as by a 5-HT_1A_ receptor antagonist [3]. 

**Figure 1:**
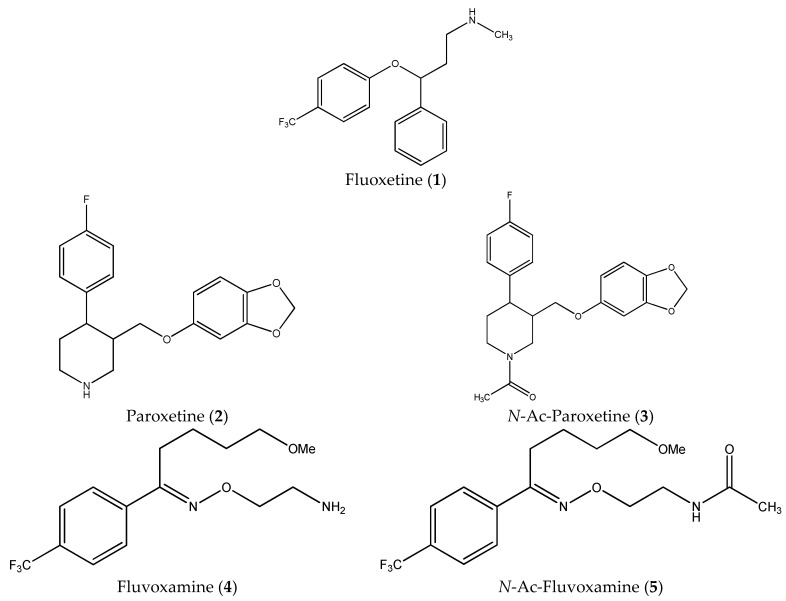
Structures of fluoxetine (**1**), paroxetine (**2**), fluvoxamine (**4**) and acetylated derivatives of paroxetine (**3**) and fluvoxamine (**5**).

The clinically used SSRIs paroxetine (**2**) and fluvoxamine (**4**) (Figure 1) were found to induce apoptosis in some cancer cell lines. It was argued that their antiproliferative activity could not be connected to serotonergic pathway since their acetylation abrogated the ability of these compounds to inhibit 5-HT uptake, but did not impair their cytotoxic activity (it was also suggested that the compounds are unlikely to represent useful lead compounds for inducing apoptosis in B-cell-derived tumors) [4,5].

**Figure 2:**
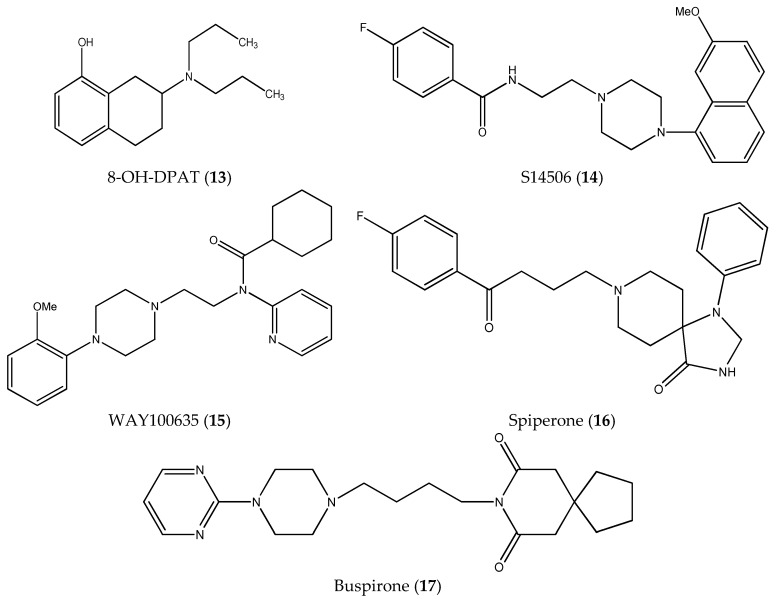
Structures of 5-HT_1A_ receptor agonists (**13**, **14**, and **17**), antagonist (**15**) and inverse agonist (**16**).

In this paper, we examined the cytotoxic activity of several compounds endowed with serotonergic activity including known compounds—fluoxetine (**1**), paroxetine (**2**), fluvoxamine (**4**) (and acetylated derivatives of paroxetine (**3**) and fluvoxamine (**5**)) (Figure 1), compounds (**6**–**8**) from our previous studies [6] and new synthesized compounds (**9**–**12**) as serotonin transporter (SERT) inhibitors (Table 1) as well as 5-HT_1A_ receptor agonists (2*R*)-(+)-8-Hydroxy-2-(di-*n*-propylamino)tetralin hydrobromide (8-OH-DPAT) (**13**), 4-Fluoro-*N*-[2-[4-(7-methoxy-1-naphthalenyl)-1-piperazinyl]ethyl]benzamide hydrochloride (S 14506) (**14**) and antagonist *N*-[2-[4-(2-Methoxyphenyl)-1-piperazinyl]ethyl]-*N*-2-pyridinylcyclohexanecarboxamide maleate (WAY100635) (**15**) (Figure 2). The antiproliferative activity of the compounds was examined in SH-SY5Y and PC-3 cancer cell lines (with Balb/c 3T3 cells as a reference). 

## 2. Results

### 2.1. Experimental Studies

New SERT inhibitors, compounds KW117 (**6**), AZ05 (**7**), AZ07 (**8**), AB5A (**9**), AB9 (**10**), AB21 (**11**) and AB22 (**12**), possessed a nanomolar affinity for SERT (Table 1). Compounds KW117 (**6**), AZ05 (**7**) and AZ07 (**8**) exhibited moderate antidepressant activity in the in vivo Porsolt forced swim test [6]. It appeared that almost all of the examined compounds (besides 5-HT_1A_ receptor agonist 8-OH-DPAT, SSRI fluvoxamine against SH-SY5Y and AB9 against both tumor lines) exhibited moderate to high cytotoxic activity at the micromolar level against neuroblastoma and prostate cancer cells (Table 2).

Compound AZ07 (IC_50_ = 5.74 ± 1.02 μM) showed the highest absolute cytotoxicity to PC-3 prostate cancer cells, and the 5-HT_1A_ receptor agonist S14506 (IC_50_ = 4.27 ± 0.41 μM) had the highest cytotoxicity to neuroblastoma SH-SY5Y cells. Of the known SERT inhibitors, paroxetine had the highest cytotoxicity, and fluvoxamine had the lowest. Acetylation of fluvoxamine and paroxetine diminished the cytotoxicity of these derivatives (not alike in B- and T-cell derived tumors [4]) both to PC-3 (IC_50_ ratio fluvoxamine/Ac-fluvoxamine—0.52; paroxetine/Ac-paroxetine—0.27) and SH-SY5Y (IC_50_ ratio fluvoxamine/Ac-fluvoxamine—0.80; paroxetine/Ac-paroxetine—0.28) tumors. 

The influence of the examined compounds on the cyclic adenosine monophosphate (cAMP) and extracellular signal-regulated kinase 1 and 2 (ERK1/2) pathways in human embryonic kidney 293 (HEK293) cells that overexpress the *5-HT1AR* gene has also been determined. Most of the compounds exhibited either agonistic or antagonistic activity on both the cAMP and MAPK/ERK pathways (Table 3). However, some of them—AB22 (**8**), paroxetine (**2**) and Ac-paroxetine (**3**)—behaved similar to mixed agonists–antagonists, exhibiting antagonistic activity on the cAMP pathway and agonistic activity on the MAPK/ERK pathway. 

**Figure 3:**
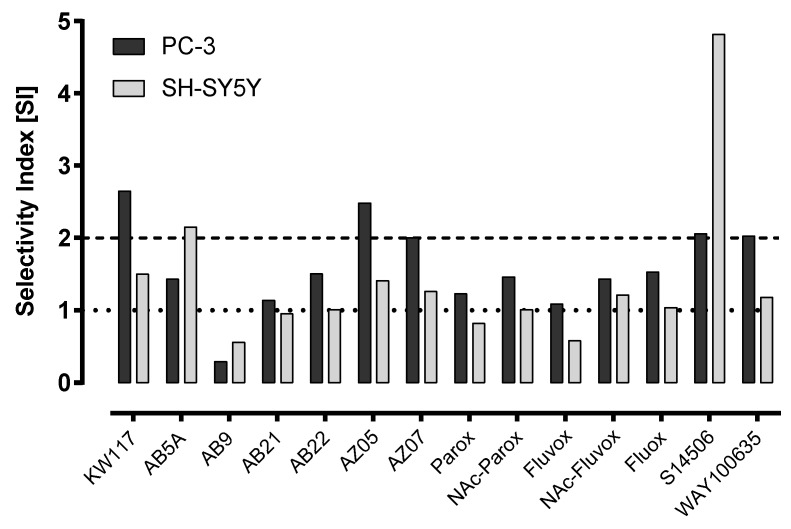
Selectivity index (SI = IC_50_ for normal cell line/IC_50_ for tumor cell line).

It is reasonable to express the cytotoxic activity of a compound against cancer cells as a selectivity index (SI) calculated as (IC_50_ control cells/IC_50_ cancer cells) (normal murine Balb/c 3T3 fibroblast cells were used as a control, Table 2, Figure 3). It is assumed that a compound with SI ≥ 2 may be considered a potential anticancer drug, since a high SI value reflects selectivity towards cancer cells while a SI value < 2 reflects general toxicity [7]. The compounds that had the desired SI values against SH-SY5Y cells were S14506 (SI 4.82) and AB5A (SI 2.15); those with desired SI values against PC-3 cells were S14506 (SI 2.06), WAY100635 (SI 2.03), KW117 (SI 2.64), AZ05 (SI 2.48), and AZ07 (SI 2.00). Only compound S14506 had a SI higher than 2 in both cell lines. For both tumor lines, lack of selective cytotoxicity showed AB9. Compound S14506 had the highest absolute activity against neuroblastoma tumor cells, and compound AZ07 had the highest absolute activity against PC-3 prostate cancer cells. 

**Figure 4:**
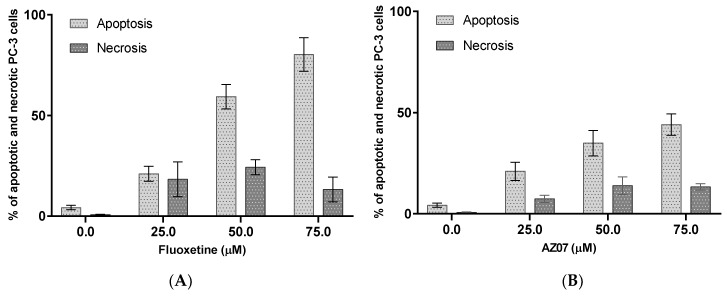
Induction of apoptosis (sum of early and late apoptosis) and necrosis in PC-3 prostate cancer cells after 72 h of incubation with fluoxetine (**A**) and compound AZ07 (**B**).

The ability of on fluoxetine (**1**, commonly used antidepressant drug) and compound AZ07 (**8**) to induce apoptosis as a cytotoxic mechanism was evaluated. Fluoxetine dose-dependently induced substantial apoptosis in PC-3 prostate cancer cells (Figure 4, 21, 60, 80% apoptosis at 25, 50 and 75 μM respectively, cytotoxic activity against PC-3 cells IC_50_ = 17.10 µM). The proapoptotic activity of compound AZ07 (exhibiting the highest absolute cytotoxicity against prostate cancer cells, IC_50_ = 5.74 µM) was almost twice smaller even if it was examined at 75 μM (20, 35, 44% apoptosis at 25, 50 and 75 μM respectively). This result could suggest that, in the case of compound AZ07 (maybe in other cases as well), the cell death may be induced through pathways other than apoptosis [8].

**Figure 5:**
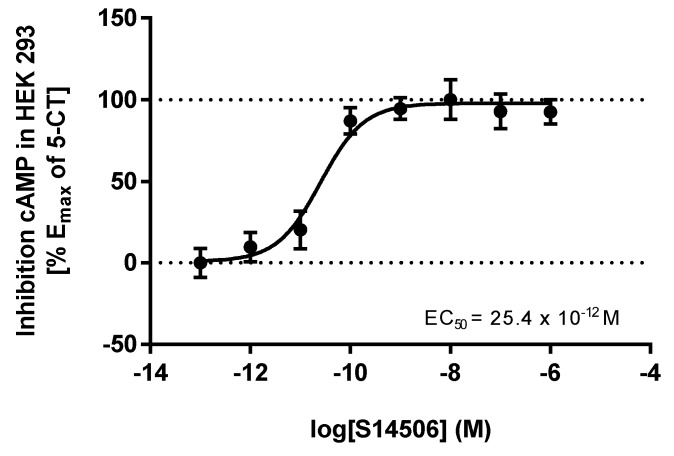
Influence of S14506 on the (1 µM) forskolin-stimulated cAMP level in HEK293 cells that overexpress the *5-HT1AR* gene.

**Figure 6:**
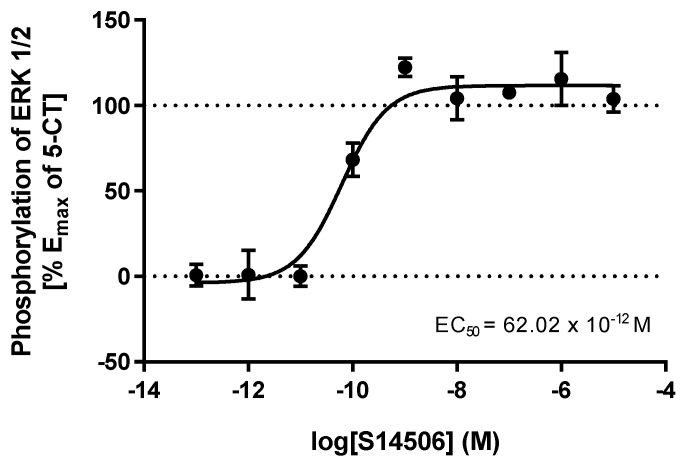
Influence of S14506 on pERK1/2 level in HEK293 cells that overexpress the *5-HT1AR* gene.

**Figure 7:**
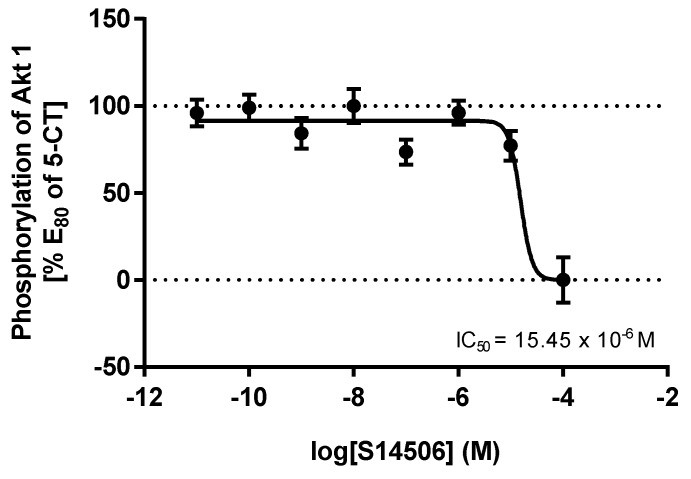
Influence of S14506 on pAkt level in HEK293 cells that overexpress the *5-HT1AR* gene.

S14506, an atypical selective agonist of the 5-HT_1A_ receptor, demonstrated significant inhibitory effects on PC-3 cells (IC_50_ = 9.98 ± 1.26 μM), and had the highest activity on SH-SY5Y cells (4.27 ± 0.47 μM). In HEK293 cells that overexpress the *5-HT1AR* gene, it was found that S14506 acted as a cAMP pathway agonist (dose-dependently diminishing cAMP levels, EC_50_ = 25.4 pM) and a MAPK/ERK1/2 pathway agonist (elevating phosphorylated ERK1/2 levels, EC_50_ = 93.0 pM) (Figure 5 and Figure 6). It should be noted, however, that S14506 was found to be an Akt pathway antagonist in HEK293 cells that overexpress the *5-HT1AR* gene (Figure 7). Akt may activate nuclear translocation of NF-κB, leading to caspase-3 inhibition and cell survival. The prosurvival activity of Akt may be reversed by Akt antagonists [9,10]. Therefore, the antagonistic activity of S14506 on Akt may induce caspase-3 activity and cytotoxicity. 

**Figure 8:**
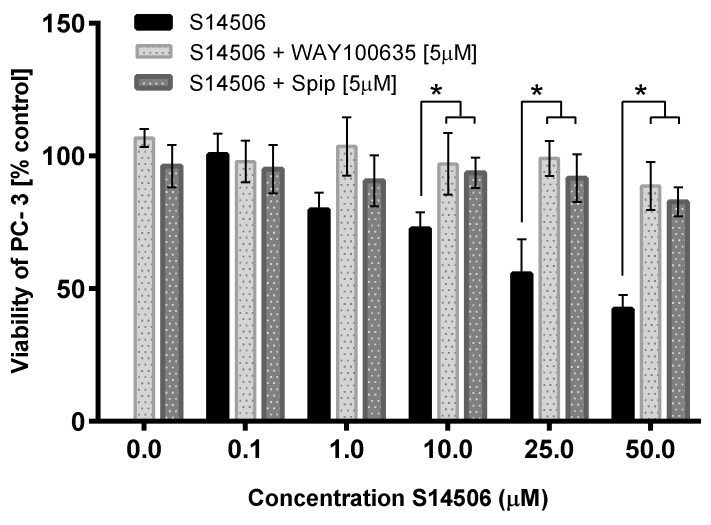
Cytotoxicity of S14506 on PC-3 cells in the presence of WAY100635 (5 µM) and spiperone (5 µM). * *p* < 0.05 vs. control.

**Figure 9:**
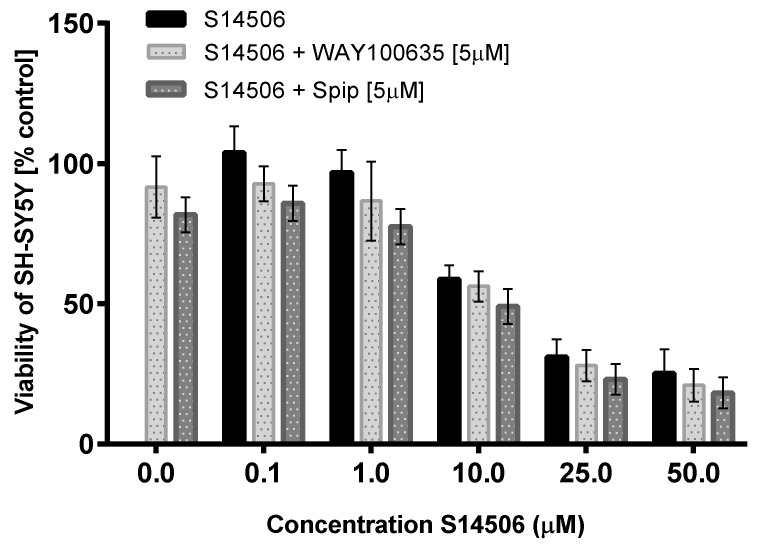
Cytotoxicity of S14506 on NH-SY5Y cells in the presence of WAY100635 (5 µM) and spiperone (5 µM).

The cytotoxic activity of S14506 against prostate cancer PC-3 cells (but not against neuroblastoma SH-SY5Y cells, Figure 8 and Figure 9) was reversed by treatment with the 5-HT_1A_ receptor antagonist WAY100635 and inverse agonist spiperone. 

**Figure 10:**
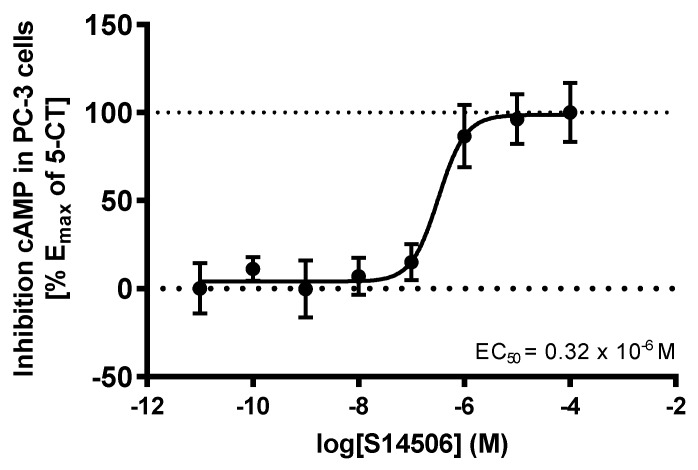
Influence of S14506 on the cAMP level in PC-3 prostate cancer cells (1 µM forskolin).

It was also found that S14506 activated the cAMP biochemical pathway in PC-3 cells (IC_50_ = 0.32 µM, Figure 10) but not in SH-SY5Y cells.

Compound S14506, although structurally related to the 5-HT_1A_ receptor inverse agonist spiperone, has been found to be one of the most potent agonists of the receptor, with very high affinity (K_d_ = 0.79 ± 0.2 nM, compared to 8-OH-DPAT K_d_ = 1.5 ± 0.5 nM). Additionally, the affinity of S14506 (but not of 8-OH-DPAT) was reduced by divalent manganese, magnesium and calcium ions. The presence of sodium ions markedly reduced the binding of 8-OH-DPAT but not the binding of S14506 [11]. S14506 potently reduced the duration of immobility in the forced swim test in rats at the minimal effective dose (MED) 0.01 mg/kg, s.c. (MED for 8-OH-DPAT was 0.63 mg/kg). The action of S14506 was blocked by the 5-HT_1A_ receptor antagonist WAY100135. It was proposed that the antidepressant action of the compound is conveyed by postsynaptic 5-HT_1A_ receptors [12]. It was also found that compound S14506 exhibited the properties of a dopamine D2 receptor antagonist [13].

### 2.2. Molecular Modelling

**Figure 11:**
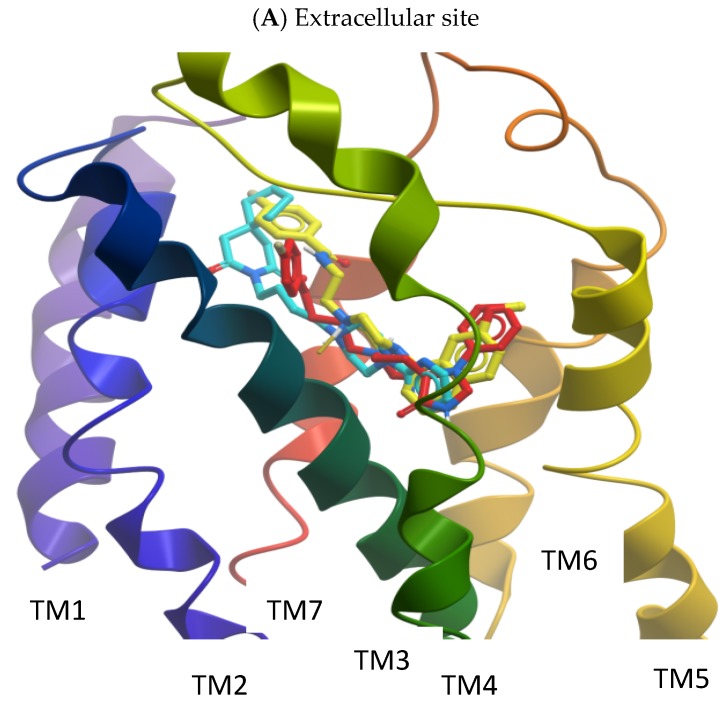

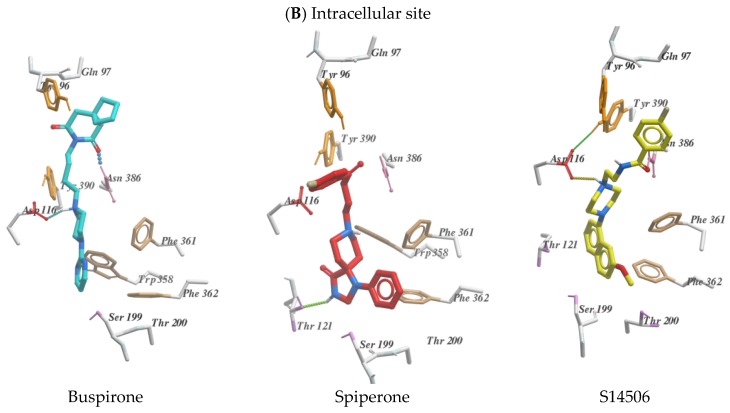
(**A**) The binding pose of S14506 (yellow), buspirone (cyan) and spiperone (red) in the 5HT_1A_ receptor model (colored from *N*-terminal (violet) to C-terminal (red)) and main interactions in the binding pocket of the 5-HT_1A_ receptor; (**B**) interaction of S14506, buspirone and spiperone with the residues in the 5-HT_1A_ binding pocket.

After docking, the ligands (buspirone, S14506, and spiperone) bound in a similar mode to the pocket formed by transmembrane helices (TM): TM3, TM5, TM6 and TM7 (Figure 11). The binding energies for buspirone, S14506 and spiperone were similar: −19.46, −22.46 and −21.21 kcal/mol, respectively. The charged piperazine nitrogen atom of the compounds interacted with residue Asp116 in TM3, which is the key recognition site for monoamine G-protein coupled receptor (GPCR) ligands [14]. The docking studies indicated that buspirone binds to the 5-HT_1A_ receptor model in a similar manner as described earlier [15]. Interactions between the pyrimidine moiety of buspirone and TM3, TM5, TM6 were observed. The azaspirone portion of buspirone was close to TM2 and TM7, forming a hydrogen bond with Asn386 in TM7. Compound S14506, similar to buspirone, interacted with the 5HT_1A_ receptor model at transmembrane helices TM3, TM5, TM6, and TM7 as well as with the extracellular loop 2 (ECL2) (Figure 11). S14506 is in a position with the naphthyl moiety located inside the binding pocket of the 5HT_1A_ receptor and the fluoro group atom near the extracellular part of the receptor. The S14506 flouro group interacts with amino acids from TM2 only, and the amido moiety can form a hydrogen bond with Asn386 in TM7 in the extracellular part of the receptor. The S14506 methoxy group interacts with Ser199 and Thr200 in TM5. The aromatic interactions of the naphthyl part of S14506 with residues Phe361 and Phe362 in TM6 were also observed. The close interactions of spiperone with Asn386 in TM7 and Thr121 in TM5 were observed. In addition, spiperone and S14506 are located closer to both phenylalanines in TM6 than buspirone. Site-directed mutagenesis indicated that the affinity of aryloxypropanolamines was strongly affected by the Asn386Val mutation and that the oxygen atoms of the oxypropanol moiety are essential for the interaction with the 5-HT_1A_ receptor [16]. The hydroxyl group present in the chemical structure of many neurotransmitters e.g., serotonin, appears to form hydrogen bonds with a series of Ser/Thr residues in TMH5 [17]. A serine (Ser199) and threonine (Thr200) residue in TMH5 of the 5-HT_1A_ receptor were both capable of forming hydrogen bonds with the 5-hydroxy group of 5-HT. The importance of Thr200 for 5-HT binding was supported by site-directed mutagenesis studies [18]. Aromatic residues such as Phe361 and Phe362 in TM6 are part of an aromatic cluster that most likely regulates receptor activation [19].

It should be noted that somewhat different results were previously obtained by Milligan et al. [11], which is not surprising since the homology model of the 5-HT_1A_ receptor based on the electron-crystallographic structure of bacteriorhodopsin was used [20]. The only interactions observed in both docking studies were between the S14506 methoxy group and Ser199 and Thr200 of the 5HT_1A_ receptor models. The fluoro group was directed to the intracellular part of the receptor binding pocket, and the naphthyl moiety was directed to the opposite (extracellular) part. The fluoro group interacted with the Asn396 side chain in TM7 that, together with other residues, forms a part of the Asp-Arg-Tyr (DRY) signaling microdomain located in the intracellular part of the receptor (Asp133^3.49^, Arg134^3.50^, Asn396^7.49^). It was suggested that S14506 spans between the 5-HT recognition site and the DRY domain and may activate the interaction of the receptor with G proteins. On the basis of their results, Milligan et al. [11] suggested that the binding of [3H]-S14506 to the 5-HT_1A_ receptor appears to be more similar to that of an antagonist, or an inverse agonist, than that of an agonist and that the efficacy of the drug at the receptor may be a consequence of a direct interaction with the signaling residues.

## 3. Discussion

It should be noted that several SSRIs (as well as some 5-HT_1A_ receptor antagonists) consistently exhibit cytotoxic and proapoptotic activity against different cancer cell lines. The blockade of SERT in general results in 5-HT transport inhibition into the presynaptic neuron, thus elevating serotonergic function. The growth stimulatory effect of 5-HT has been described in different cell types and carcinomas, and 5-HT is thought to participate in the proliferation of different cancer types; moreover, known 5-HT_1A_ receptor agonists were not found to exhibit cytotoxic activity in cancer cells (for a review, see [2]). The activity of 5-HT was shown to be connected to biochemical pathways such as those of MAPK(MEK)/ERK, Akt and NF-κB. It should be noted that NF-κB (formed as a dimer of specific subunits), by coordinating different subunits in the formation of a dimer, may regulate the transcription of different genes including those relating to apoptotic, antiapoptotic and inflammatory processes; the effects of NF-κB on cell survival or death will depend on the activation of distinct NF-κB factors [21,22]. It was suggested that the activation of different serotonergic pathways and the activation or blockade of 5-HT_1A_ receptors may result in either prosurvival or proapoptotic activity [15,23,24]. Additionally, considering that several biochemical pathways may be directed by the 5-HT_1A_ receptor [25], one should not expect a straightforward relationship between the serotonergic activity of SSRIs and 5-HT_1A_ receptor ligands and their cytotoxic or cytoprotective activity. 

Our results appear to support such an assumption. Most of the compounds (**1**–**15**) exhibited either agonistic or antagonistic activity on both the cAMP and pERK1/2 pathways in HEK293 cells that overexpress the *5-HT1AR* gene. However, some of the compounds (**2**, **3** and **12**) behaved similarly to mixed agonist–antagonists, exhibiting antagonistic activity on the cAMP pathway and agonistic activity on the ERK1/2 pathway. This result suggests that the cytotoxic activity of the compounds examined may be dependent on the activation of different biochemical pathways.

It was found that compound AZ07 had higher cytotoxic activity than that of fluoxetine in prostate PC-3 cancer cells. It should be noted, however, that fluoxetine had high proapototic activity and that compound AZ07 had low proapototic activity in PC-3 cancer cells. This observation suggests that the cytotoxic activity of compound AZ07 may be dependent on mechanisms other than apoptosis [26]. 

The acetylation of fluvoxamine and paroxetine diminished their cytotoxic activity (unlike in B- and T-cell-derived tumors [4]) both on PC-3 (IC_50_ ratio fluvoxamine/Ac-fluvoxamine—0.52; paroxetine/Ac-paroxetine—0.27) and SH-SY5Y (IC_50_ ratio fluvoxamine/Ac-fluvoxamine—0.80; paroxetine/Ac-paroxetine—0.28) cells. 

The cytotoxic activity of compound S14506, an 5-HT_1A_ receptor agonist, was consistently reversed by 5-HT_1A_ receptor antagonists (WAY100635 and spiperone) in prostate but not in neuroblastoma cancer cells. Additionally, it was found that compound S14506 is an agonists of the cAMP and ERK1/2 pathways exhibited but is an antagonist of the prosurvival Akt pathway, which would explain its cytotoxic activity. This result suggests that either the cytotoxic activity of S14506 may be connected to the activation of the 5-HT_1A_ receptor biochemical transduction pathway in the prostate, but not in neuroblastoma cancer cells, or that a receptor antagonist may be transduction pathway-specific.

Combining our results (particularly those concerning the cytotoxicity of and the biochemical pathways activated by compounds **8**, **14** and the acetylated paroxetine and fluvoxamine derivatives) with the results of Schuster et al. [4] suggests that the mechanism of a compound’s cytotoxic activity (serotonergic against nonserotonergic) may depend on the compound and cancer type being examined. 

On the basis of the S14506 previous docking studies to the bacteriorhodopsin homology-based 5-HT_1A_ receptor, model microenvironments of the receptor activation made by the hydrophilic-conserved residues of the adjacent Arg3.50, Asp/Glu3.49, Asn1.50, Asp2.50, Asn7.49 and Tyr7.53 were identified [11]. It was also suggested that the compound may bind preferentially to an uncomplexed receptor (whereas 8-OH-DPAT binds preferentially to a receptor with prebound trimeric G protein), thus following the classic scheme for the binding of inverse agonists (spiperone, a 5-HT_1A_ receptor inverse agonist, binds preferentially to the free receptor rather than to the coupled receptor). The experimental data indicate that S14506 has preferential affinity for the non-functional antagonist state of the receptor, but after binding, the S14506-receptor complex signals the G protein more efficiently than do other ligand-receptor complexes [11]. Our results suggest that S14506, buspirone and spiperone occupy a similar binding pocket and interact with similar 5-HT_1A_ receptor residues. We did not observe the interaction of either compound with the conserved DRY microdomain. It is therefore difficult to determine if the S14506 binding mode is agonist- or antagonist-like. Although our arrangement of S14506 in the 5-HT_1A_ receptor model is different to that of Milligan et al. [11] (different templates were used for homology model construction), the ligand occupies more or less the same binding pocket. Yin et al. [27] proposed that ring-like groups of inverse agonist/antagonists shift towards TM6 relative to agonists, leading to the outward movement of TM6, coupled with an inward shift of the intracellular end of this helix. According to our simulations, spiperone and S14506 are located closer to both phenylalanines in TM6 than buspirone, exhibiting a more antagonist-like binding mode. By interacting with the aromatic residues in TM6, the compounds could form an inactivating complex, but both S14506 and buspirone are cAMP pathway agonists (S14506 was also found to be an agonist of the ERK1/2 pathway). It should be noted that S14506 was found to have antagonistic activity on the Akt pathway.

## 4. Materials and Methods

### 4.1. Compounds

Fluvoxamine (**4**) and paroxetine (**2**) were isolated from their dosage forms Fevarin (Abbott Healthcare Products B.V., Weesp, The Netherlands) and Rexetin (Gedeon Richter Plc., Budapest, Hungary), respectively. Tablets were crushed into powder, and the salts contained in the tablet mass (fluvoxamine maleate and paroxetine hydrochloride) were converted into their free base form (15% NaOH aq); then, the aqueous mixtures were extracted with chloroform, and extracts were purified by silica gel flash chromatography (9:1, chloroform/methanol). Acetylated derivatives of fluvoxamine (**5**) and paroxetine (**3**) were obtained by acetylation with acetic anhydride according to a previously published procedure [4]. Fluoxetine (**1**) was purchased from Sigma-Aldrich, St. Louis, MO, USA. 8-OH-DPAT (**13**), S14506 (**14**), WAY100635 (**15**) and spiperone (**16**) were purchased from Tocris Bioscience, Bristol, UK.

KW117 (6-nitro-2-(4-octylpiperazin-1-yl)quinoline hydrochloride) (**6**), AZ05 (6-nitro-2-(4-nonylpiperazin-1-yl)quinolone hydrochloride) (**7**), and AZ07 (6-nitro-2-(4-undecylpiperazin-1-yl)quinoline hydrochloride) (**8**) were obtained as described in Gabrielsen et al. [6]. New compounds: AB5A (2-[4-(4-fluorobenzyl)piperazin-1-yl]-6-nitroquinoline hydrochloride) (**9**), AB9 (6-nitro-2-[4-(2-phenylethyl)-piperazin-1-yl]-6-nitroquinoline hydrochloride) (**10**), AB21 (2-[4-(cyclohexylmethyl)piperazin-1-yl]-6-nitroquinoline hydrochloride) (**11**), and AB22 (2-[4-(cyclobutylmethyl)piperazin-1-yl]-6-nitroquinoline hydrochloride) (**12**) were synthesized as described below in Section 6—Experimental procedures.

### 4.2. Cell Lines

#### 4.2.1. PC-3 and SH-SY5Y, as well as HEK293 and Balb/c 3T3 Cells as a Reference

SH-SY5Y is a human neuroblastoma-like cell line often used as an in vitro model to study the function and differentiation of neurons. These cells produce serotonin and express major serotonergic proteins and mRNA including receptor 5-HT_1A_ [28]. PC-3 is an androgen-independent neuroendocrine tumor line, does not form glands, is negative for androgen receptor (AR), prostate-specific antigen (PSA) and expresses serotonergic receptor subtype 1 (5-HTR_1A_) [29]. PC-3 is more diverse and aggressive than the DU-145 prostate cancer cell line. 

Transformed human embryonic kidney cells (HEK293) were used to determine the functional activity of 5-HT_1A_ receptor ligands. The Balb/c 3T3 cell line is a standard cell line often used as a model in studies of normal fibroblast cells. Lines were obtained from American Type Culture Collection (ATCC) Manassas, VA, USA.

#### 4.2.2. Preparation of a Stable Transfectant 5-HT_1A_—HEK293 Cell Line

The transfectant’s parent line, Flp-In T-Rex-293, is a HEK293 (CRL-1573) cell line that was obtained from ATCC. Flp-In T-Rex-293 cells (Cat. No. R780-07), which stably express the lacZ-Zeocin gene fusion and Tet repressor with regulatory pcDNA6/TR plasmid, are part of the System (Cat. No. K6500-01) Life Technology (Carlsbad, CA, USA).

Lines were obtained by cotransfection with a pcDNA5/FRT/TO plasmid containing the gene encoding the serotonin receptor 5-HT_1A_ and a pOG44 vector containing Flp recombinase. The Flp recombinase plasmid inserts the 5-HT_1A_ gene into the genome at Flp Recombination Target (FRT) sites. After the cultivation of stable lines, gene expression can be induced by the addition of tetracycline. The enhanced green fluorescent protein (EGFP) sequence was also introduced into the system next to the appropriate receptor protein. Ligations were performed using a ligase from New England Biolabs (T4 DNA Ligase M0202S, Ipswich, MA, USA).

Transformations were performed with One Shot^®^ TOP10 chemically competent *E. coli* C4040-10, Life Technologies. Selection of clones possessing the plasmid was carried out using their antibiotic (ampicillin) resistance. The resulting clones were checked for the presence of a recombinant gene by sequencing. Transfections were performed using Lipofectamine^®^ 3000 transfection reagent (L3000-008, Life Technologies, Carlsbad, CA, USA). Antibiotic selection was performed using hygromycin and blasticidin.

5-HT_1A_ receptor expression in the selected 10 clones of cells transfected with different plasmids was confirmed by Western blot. The level of expression and cellular localization of receptor proteins was monitored by immunofluorescence or detection by sodium dodecyl sulfate polyacrylamide gel electrophoresis (SDS-PAGE) protein electrophoresis. Enhanced Green Fluorescent Protein (EGFP) fluorescence allowed measurement and visualization.

### 4.3. Cell Cultures

Cultures of PC-3 cells were grown in RPMI 1640 medium (Sigma-Aldrich, St. Louis, MO, USA), Balb/c 3T3 in Dulbecco’s modified Eagle’s medium (DMEM), SH-SY5Y in DMEM: F12 (Sigma-Aldrich, St. Louis, MO, USA), and HEK293 cells in DMEM (Gibco, Grand Island, NY, USA). All cell lines were grown at 37 °C in a humidified atmosphere with 5% CO_2_. All media contained 1% GlutaMax (Gibco, Grand Island, NY, USA), 1% Antibiotic Antimycotic Solution (10,000 units/mL penicillin G, 10 mg/mL streptomycin sulfate, 25 mg/mL amphotericin B, Sigma-Aldrich, St. Louis, MO, USA) and 10% heat-inactivated and sterile-filtered fetal bovine serum (FBS) (Gibco, Grand Island, NY, USA), except Balb/c 3T3 cells, whose media was supplemented with 10% calf serum (Biowest, Courtaboeuf, France).

Cell lines were passaged every 3–4 days by trypsinization with 0.25% trypsin solution, with or without EDTA (2–5 min) (Thermo Fisher Scientific, Waltham, MA, USA). Cells used for experiments were derived from a continuous culture and were in the exponential growth phase. The cells were counted using a Handheld Automated Cell Counter – Scepter (Merck Millipore, Billerica, MA, USA). 

### 4.4. Study of In Vitro Binding Affinity of Compounds for Serotonin Transporter (SERT)

The experiments were performed according to the method described by Owens et al. [30] Rat cerebral cortex was homogenized in 30 volumes of ice-cold 50 mM Tris-HCl containing 150 mM NaCl and 5 mM KCl, pH 7.7 at 25 °C and centrifuged at 20,000 × *g* for 20 min. The supernatant was decanted, and the pellet was resuspended in 30 volumes of buffer and centrifuged three times. The final pellet was suspended in Tris-HCl buffer at a proportion of approximately 5 mg wet weight/mL. An increasing concentration of the analyzed compounds (30 μL) was added to the incubation mixture containing 240 μL of tissue suspension and 30 μL of 1 nM [^3^H]-citalopram (used for labeling 5-HT-transporter). Non-specific binding was determined in the presence 30 μL of 1 μM imipramine (displacer). Duplicate samples were incubated at room temperature for 1 h. The incubation was terminated by rapid filtration over fiberglass filters FilterMate B (Perkin Elmer, Waltham, MA, USA) using a 96-well FilterMate harvester (PerkinElmer, Waltham, MA, USA). The radioactivity of the filters was measured with a MicroBeta TriLux 1450 scintillation counter. *K_i_* values were calculated from the Cheng–Prusoff equation [31]:

(1) $$K_{i}=\frac{{\mathrm{IC}}_{50}}{1+\frac{Lo}{K_{D}}}$$

where *Lo* is the labeled ligand concentration, and *K_D_* is the dissociation constant of the labeled ligand.

### 4.5. Examination of the Functional Activity of 5-HT_1A_ Receptor Ligands in Transformed HEK293

The examination of the functional activity read out as cAMP level inhibition and ERK1/2 and Akt1 phosphorylation was performed according to the instructions of the One-plate Cisbio Bioassays cAMP dynamic 2, phospho-ERK (Thr202/Tyr204) and Akt1 phospho- S473 Cellular Assay Kits respectively, using a HEK293 human fibroblast cell line that overexpresses 5-HT_1A_ receptors. The measurements were performed in 384-well microtiter plates using 200,000, 1,875,000 or 2,000,000 cells/mL. The cAMP levels in PC-3 cells were determined using 600,000 cells/mL. Receptor activation with the appropriate ligands was carried out for 30 min, and then the cells were lysed. The results were analyzed with GraphPad Prism v 6.0 (GraphPad Software, San Diego, CA, USA) using its in-built functions. Ligand activity was determined using a 4-parameter equation of the dose-response function. The response curves were obtained by plotting the HTRF^®^ ratio (signal ratio 665/620 nm × 10,000) relative to the log concentration of the [compound] M.

All compounds were tested with both agonists and antagonists. To test the response of cAMP to an agonist the cells were activated with forskolin (1 µM) followed by agonist addition. Antagonist activity (both for cAMP and pERK1/2 determination) was measured against the reference agonist (5-carboxamidotryptamine)-pretreated cells (0.1 μM of 5-CT = EC_80_, for cAMP measurements, forskolin-stimulated cells were used) and expressed as IC_50_ (antagonist concentration showing 50% inhibition of maximal response). Data are an average of minimum three independent experiments in duplicate. The control of specific activity was measured under the same conditions on cells lacking the 5-HT_1A_ receptor. The results were expressed as % of the reference agonist activity. In the antagonist mode 100% of the reference agonist activity (EC_80_) corresponded to 0% antagonist activity.

### 4.6. Determination of the Effect of Test Compounds on Cell Survival

The effects of the test compounds on the survival of PC-3, SH-SY5Y, Balb/c 3T3 and HEK293 cells was measured with the aid of the 3-(4,5-dimethylthiazol-2-yl)-2,5-diphenyltetrazolium bromide (MTT) assay. The cells were cultured in 96-well plates (100 μL/well) for 72 h. The optimized initial densities were the following: for PC-3—60,000 cells/mL, SH-SY5Y and Balb/c 3T3—50,000 cells/mL. The concentration of the starter culture was determined in a preliminary experiment to provide optimum cell growth during the experiment, and to ensure continuous exponential growth of cells and prevent overcrowding throughout the experimental incubation, 70% confluence was determined to be optimal for a 72 h test.

The activity of the compounds was determined at the following concentrations: 0.01; 0.1; 1.0; 2.5; 5.0; 10; 15; 25; 50; 75 and 100 μM (additionally 7.5 μM for compound AB9). After 20 h preincubation and evaluation of the uniformity of cell growth, the medium was aspirated, and the respective solutions of the substances were added to the culture medium. Control cells were only treated the solvent used for the compounds and medium alone. The positive control of cytotoxicity for the control of the system was fluoxetine.

The MTT assay is a colorimetric method that measures cell viability by revealing the metabolic activity of cells in culture. MTT (3-(4,5-dimethylthiazol-2-yl)-2,5-diphenyltetrazolium bromide) is reduced by intracellular dehydrogenase, which is present only in live cells (mitochondria), to purple, water-insoluble formazan. After it is dissolved, formazan absorbance of the resulting solution is directly proportional to the number of living cells in culture. 

After appropriate incubation, the culture medium was removed. Next, 50 μL of MTT solution (0.5 mg/mL) diluted in PBS to 0.5 mg/mL was added to each well. The plates were incubated for 3 h at 37 °C. Then, the precipitated formazan was dissolved by the addition of 200 μL isopropanol. After a 24-hour incubation at 4 °C in the dark, the absorbance of the dissolved formazan was measured at 570 nm (the maximum absorption of formazan) and at 690 nm (reference wavelength) using a Power Wave XS microplate spectrophotometer (Bio-Tek, Winooski, VT, USA). The plates were sealed to reduce evaporation before incubation. The test was performed in three to five independent experiments with four times the measured concentration tested. The cell survival percentage was determined as % of controls, which were cells treated with the test compound solvent at the highest concentration. The MTT data were analyzed to determinate the IC_50_ values (concentration required to reduce the viability of cells by 50% compared with the control cells) of each compound.

### 4.7. Apoptosis Determination

Apoptosis was measured using the FITC Annexin V Apoptosis Detection Kit I (BD Biosciences, San Jose, CA, USA). Cells, after incubations 72 h with tested agents, were centrifuged, rinsed twice with cold phosphate-buffered saline (PBS) and suspended in binding buffer at 1 × 10^6^ cells/mL. Then, 100-μL volumes of the cell suspensions were labeled according to the manufacturer’s instructions. Flow cytometry analysis was performed within 1 h after labeling.

### 4.8. Flow Cytometry

Flow cytometry analyses was run on a FACSCanto II flow cytometer (BD Biosciences, San Jose CA, USA) and analyzed using BD FACSDiva software. 

### 4.9. Molecular Modeling

The homology model of the 5-HT_1A_ serotonin receptor is based on the X-ray structure of the serotonin 1B receptor, (PDB ID 4IAR), and the crystal structure was employed [15,26,32]. The start and end positions of the transmembrane helices (TM) in the models were found to be the following: Val31-Val51 (TM1), Leu78-Leu99 (TM2), Gly105-Cys128 (TM3), Ser158-Ser168 (TM4), Asp192-Val213 (TM5), Met351-Val367 (TM6), and Leu380-Leu395 (TM7). 

The structures of the compounds (buspirone, S 14509 and spiperone) were taken from the PubChem database. The ligands were protonated at the nitrogen atom in the piperazine ring. ICM/Molsoft software was used for docking studies [23]. Ligands were docked to 5HT_1A_-serotonin receptor models using the usual rigid protein-flexible ligand approach [23]. The binding poses were evaluated with an all-atom ICM ligand binding score calculated using the VLS (virtual ligand screening) scoring function [33]. The VLS scoring function is the sum of the internal force field energy of the ligand and the ligand-receptor interaction energy that includes several weighted terms: van der Waals term, a hydrophobicity term, an electrostatic solvation term, hydrogen bonding interactions, and an entropic term proportional to the number of flexible torsions in the ligand.

### 4.10. Data Analysis

All experiments were performed in triplicate, and the results were obtained from at least three independent experiments. The data are expressed as the mean ± SEM (standard error of the mean). The statistical significance was calculated using an unpaired *t*-test. Differences were considered statistically significant at *p* ≤ 0.05. The experimental data were analyzed using GraphPad Prism 6.01 for Windows (GraphPad Software, San Diego, CA, USA, www.graphpad.com).

Curve fitting and calculation of IC_50_ values was performed by the GraphPad Prism program from the best-fit (R2 > 0.95) of the Hill slope curve to experimental data using nonlinear regression analysis according to the following formula:

(2) $$Y=\frac{100}{1}+10^{\left(\left({\mathrm{LogIC}}_{50}-X\right)*\;\mathrm{Hill}\;\mathrm{slope}\right)}$$

where *X* is the log of dose, *Y* is the growth inhibition value normalized to the control, and Hill slope is the unitless slope factor or Hill slope. 

## 5. Conclusions

The cytotoxic activity of several SERT inhibitors and 5-HT_1A_ receptor ligands was examined. Almost all of the examined compounds (except for 5-HT_1A_ receptor agonists 8-OH-DPAT and 5-CT, but including agonist S14506) exhibited cytotoxic activity against neuroblastoma and prostate cancer cells. Compound AZ07 had the highest absolute cytotoxic activity against PC-3 prostate cancer cell lines (IC_50_ = 5.74 μM) and the 5-HT_1A_ receptor agonist S14506 had the highest cytotoxicity against neuroblastoma SH-SY5Y cells (IC_50_ = 4.27 μM). Compound AZ07 in amounts up to 75 µM did not exhibit substantial proapoptotic activity, which could suggest that its cytotoxic activity may be connected to a different pathway. Only compound S14506 (**14**) had a SI higher than 2 in both cell lines. The highest activity against neuroblastoma tumor and androgen-insensitive human prostate cancer was found to be with compound S14506 and compound AZ07 (**8**), respectively. Therefore, further studies for compounds S14506 and AZ07 for their potential use in the prevention and treatment of cancer are needed. Most of the compounds (**1**–**15**) exhibited either agonistic or antagonistic activity on both the cAMP and pERK1/2 pathways in HEK293 cells that overexpress the *5-HT1AR* gene. However compounds **2**, **3** and **12** exhibited antagonistic activity on the cAMP pathway and agonistic activity on the ERK1/2 pathway. Thus it maybe suggested that the cytotoxic activity of the examined compounds may be connected to the activation of a particular biochemical pathway. In spite of the higher cytotoxicity of compound AZ07 compared to that of fluoxetine, the proapoptotic activity of fluoxetine was high, while the activity of compound AZ07 was low, in prostate PC-3 cancer cells. This finding suggests that the cytotoxic activity of compound AZ07 may be dependent on a mechanism different than apoptosis. Compound S14506 was found to be an antagonist of the Akt pathway. Akt may activate the nuclear translocation of NF-κB, leading to caspase-3 inhibition and cell survival. The prosurvival activity of Akt may be reversed by Akt antagonists. Therefore, the antagonistic activity of S14506 on the Akt may induce caspase-3 activity and cytotoxicity.

Combining our above results (particularly those concerning the cytotoxicity of and the biochemical pathways activated by compounds **8**, **14** and the acetylated paroxetine and fluvoxamine derivatives) with the results of Schuster et al. [4], one should not expect a straightforward relationship between the activation of particular serotonergic pathways by SSRIs and 5-HT_1A_ receptor ligands and their cytotoxic or cytoprotective activity. It was also found that NF-κB, which may be involved in 5-HT-dependent biochemical pathways by coordinating different subunits to form a dimer, may regulate the transcription of different transduction pathways, and the effects of NF-κB on cell survival or death depend on the activation of distinct NF-κB factors. Therefore, it can be suggested that the mechanism of a compound’s cytotoxic activity (serotonergic against nonserotonergic) may depend on the compound and cancer type being examined. 

The results of molecular modeling suggest that S14506, buspirone and spiperone occupy similar binding pockets and interact with similar 5-HT_1A_ receptor residues. We did not observe the interaction of either compound with the conserved DRY microdomain. S14506 and spiperone were found to be located closer to both phenylalanines in TM6 than buspirone was, exhibiting a more antagonist-like binding mode. By interacting with aromatic residues in TM6, the compounds could form inactivated complex, but both S14506 and buspirone are agonists for the cAMP pathway (and S14596 was also found to be an agonist of the ERK1/2 pathway). It should be noted that S14506 was found to be an AKT pathway antagonist.

## 6. Experimental Procedures

### 6.1. Preparation of N-Alkylaryl-6-nitroquipazines Derivatives ***9*** (AB5A) and ***10*** (AB9)

The synthesis was done according to the following scheme:

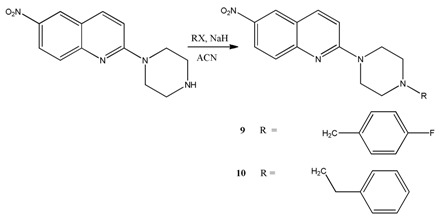



*2-[4-(4-Fluorobenzyl)piperazin-1-yl]-6-nitroquinoline* (**9**) *(AB5A).* A slurry of sodium hydride in mineral oil (100 mg, 2.47 mmol) was added to a solution of 6-nitroquipazine (0.2 g, 0.77 mmol) in acetonitrile cooled to 0 °C. After 30 min, 1-(bromomethyl)-4-fluorobenzene (0.146 g, 0.77 mmol) and 3 drops of 1,8-diazabicyclo(5.4.0)undec-7-ene (DBU) were added followed by an incubation at room temperature for 3 h. After this, the solution was filtered and evaporated under reduced pressure. The reaction mixture was purified by column chromatography (2% MeOH/CHCl_3_), yielding yellow-orange crystals.

C_20_H_19_FN_4_O_2_. M = 366.39 g/mol. Yield 60%. m.p.: 96.5–98 °C. ^1^H nuclear magnetic resonance (NMR) (300 MHz, CDCl_3_): δ 8.52 (d, 1H, *J* = 2.7 Hz, H_arom_), 8.29 (dd, 1H, *J*_1_ = 2.4 Hz, *J*_2_ = 9.3 Hz, H_arom_), 7.94 (d, 1H, *J* = 9.3 Hz, H_arom_), 7.88 (d, 1H, *J* = 9.0 Hz, H_arom_), 7.57 (m, 4H, H_benzyl_), 7.32 (dd, 1H, *J*_1_ = 5.4 Hz, *J*_2_ = 8.4 Hz, H_arom_), 3.85 (t, 2H, *J* = 4.9 Hz, CH_2_-Ph), 3.72 (t, 2H, *J* = 5.1 Hz, N(CH_2_)_piper_-Ph), 3.02 (t, 2H, *J* = 5.1 Hz, N(CH_2_)_piper_-Ph), 2.56 (t, 2H, *J* = 4.9 Hz, N(CH_2_)_piper_-CH_2_), 1.73 (s, 2H, N(CH_2_)_piper_-CH_2_). Electrospray ionisation mass spectrometry (ESI-MS) *m*/*z*: found 367.1 [M + H]^+^ (100%), calculated for [C_20_H_20_FN_4_O_2_]^+^ 367.2. 

*2-[4-(2-Phenylethyl)piperazin-1-yl]-6-nitroquinoline* (**10**) *(AB9)*. A slurry of sodium hydride in mineral oil (100 mg, 2.47 mmol) was added to a solution of 6-nitroquipazine (0.16 g, 0.62 mmol) in acetonitrile cooled to 0 °C. After 30 min, 1-(bromoethyl)- benzene (0.115 g, 0.62 mmol) and 5 drops of DBU were added, and then, the mixture was heated in a water bath under reflux for 20 h. After this step, the solution was filtered and evaporated under reduced pressure. The reaction mixture was purified by column chromatography (2% MeOH/CHCl_3_), yielding yellow-orange crystals.

C_21_H_22_N_4_O_2_. M = 362.43 g/mol. Yield 60%. m.p.: 143–145 °C. ^1^H NMR (300 MHz, CDCl_3_): δ 8.60 (d, 1H, *J* = 2.7 Hz, H_arom_), 8.25 (dd, 1H, *J*_1_ = 2.7 Hz, *J*_2_ = 9.3 Hz, H_arom_), 8.10 (d, 1H, *J* = 9.3 Hz, H_arom_), 7.61 (d, 1H, *J* = 9.3 Hz, H_arom_), 7.24 (m, 6H, H_arom_, H_phenyl_), 5.24 (d, 2H, *J* = 1.8 Hz, N(CH_2_)_piper_-Ph), 5.19 (d, 2H, *J* = 1.8 Hz, N(CH_2_)_piper_-Ph), 5.07 (d, 2H, *J* = 1.8 Hz, N(CH_2_)_piper_-CH_2_), 5.03 (d, 2H, *J* = 1.8 Hz, N(CH_2_)_piper_-CH_2_), 2.84 (t, 2H, *J* = 7.6 Hz, CH_2_Ph), 2.64 (m, 2H, NCH_2_). ESI-MS *m*/*z*: found 363.1 [M + H]^+^ (100%), calculated for [C_21_H_23_N_4_O_2_]^+^ 363.2.

### 6.2. Preparation of 6-Nitroquipazine N-Cycloalkyl Derivatives ***11*** (AB21) and ***12*** (AB22)

The synthesis was done according to the following scheme:

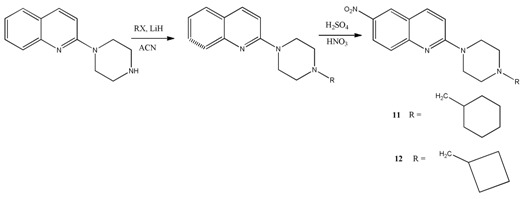



A solution of lithium hydride (100 mg, 12.5 mmol) was added to a solution of 6-nitroquipazine (100 mg, 2.47 mmol) in acetone cooled to 0 °C. After 30 min, cycloalkyl halide (1.88 mmol) and 10 drops of DBU (1,8-diazabicyclo[5.4.0]undec-7-ene) were added, and then, the mixture was heated on a water bath under reflux for 72 h. After this step, the solution was filtered and evaporated under reduced pressure. The reaction mixture was purified by column chromatography (1–4% MeOH/CHCl_3_), yielding yellow-orange crystals. The resulting N-cyclic derivative of quipazine was directly subjected to a nitration reaction. The derivative (0.71 mmol) was cooled to 0 °C and dissolved in concentrated H_2_SO_4_ (1.83 cm^3^), after which concentrated HNO_3_ (0.35 cm^3^) was added dropwise. After approximately 1 h, the mixture was poured onto crushed ice and basified with 12 M NaOH to pH = 11. The mixture was then extracted several times with methylene chloride, and the extract was dried over anhydrous MgSO_4_ for 24 h. Next, the solvent was filtered and evaporated. A pure brownish-orange-colored product was obtained by column chromatography (1–4% MeOH/CHCl_3_).

*2-[4-(Cyclohexylmethyl)piperazin-1-yl]-6-nitroquinoline* (**11**) *(AB21)*

C_20_H_26_N_4_O_2_. M = 354.45 g/mol. Yield 60%. m.p. 107–108 °C. ^1^H NMR (300 MHz, CDCl_3_): δ 8.53 (d, 1H, *J* = 2.7 Hz, H_arom_), 8.29 (dd, 1H, *J*_1_ = 2.7 Hz, *J*_2_ = 9.3 Hz, H_arom_), 7.94 (d, 1H, *J* = 9.3 Hz, H_arom_), 7.65 (d, 1H, *J* = 9.3 Hz, H_arom_), 7.05 (d, 1H, *J* = 9.3 Hz, H_arom_), 3.85 (t, 4H, *J* = 5.0 Hz, N(CH_2_)_2piper_-Ph), 2.53 (t, 4H, *J* = 4.4 Hz, N(CH_2_)_2piper_-CH_2_), 2.19 (d, 2H, *J* = 6.9 Hz, NCH_2_), 1.78 (m, 4H, 2 × (CH_2_)_cyclohexyl_), 1.53 (m, 4H, 2 × (CH_2_)_cyclohexyl_), 1.22 (m, 1H, CH_cyclohexyl_), 0.90 (m, 2H, (CH_2_)_cyclohexyl_). ESI-MS *m*/*z*: found 355.1 [M + H]^+^ (100%), calculated for [C_20_H_27_N_4_O_2_]^+^ 355.2. 

*2-[4-(Cyclobutylmethyl)piperazin-1-yl]-6-nitroquinoline* (**12**) *(AB22)*

C_18_H_22_N_4_O_2_. M = 326.39 g/mol. Yield 50%. M. m.p. 99–101 °C. ^1^H NMR (300 MHz, CDCl_3_): δ 8.53 (d, 1H, *J* = 2.7 Hz, H_arom_), 8.29 (dd, 1H, *J*_1_ = 2.7 Hz, *J*_2_ = 9.0 Hz, H_arom_), 7.95 (d, 1H, *J* = 9.3 Hz, H_arom_), 7.65 (d, 1H, *J* = 9.3 Hz, H_arom_), 7.05 (d, 1H, *J* = 9.3 Hz, H_arom_), 3.89 (t, 4H, *J* = 4.8 Hz, N(CH_2_)_2piper_-Ph), 2.64 (m, 5H, N(CH_2_)_2piper_-CH_2_, CH_cyclobutyl_), 2.52 (m, 2H, NCH_2_), 2.11 (m, 2H, CH_2cyclobutyl_), 1.82 (m, 4H, 2 x CH_2cyclobutyl_). ESI-MS *m*/*z*: found 327.1 [M + H]^+^ (100%), calculated for [C_18_H_23_N_4_O_2_]^+^ 327.2.

## Figures and Tables

**Table 1:** Structures and binding affinities of new serotonin transporter (SERT) inhibitors.

Compound	Structure	Symbol	SERT K_i_ ± SEM, nM
**6**	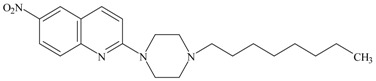	KW117	20.8 ± 3.8 [6]
**7**	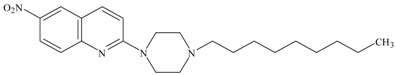	AZ05	1.8 ± 0.2 [6]
**8**	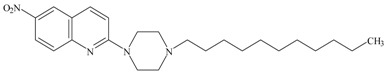	AZ07	12.6 ± 1.3 [6]
**9**	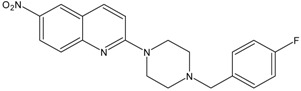	AB5A	7.4 ± 2.4
**10**	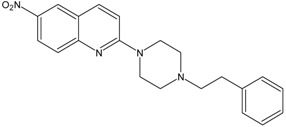	AB9	1.3 ± 0.6
**11**	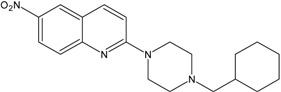	AB21	14.9 ± 1.6
**12**	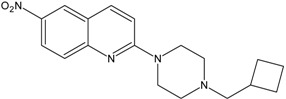	AB22	7.6 ± 0.8

SEM—standard error of the mean.

**Table 2:** Effect of new and classic selective serotonin reuptake inhibitors (SSRIs) and ligands of 5-HT_1A_ receptor on PC-3, SH-SY5Y and Balb/c 3T3 cells.

No.	Ligand	Cells	IC50 ± SEM (µM)	Selectivity Index
1.	Fluoxetine (**1**)	PC-3	17.10 ± 1.38 *	1.53
		SH-SY5Y	25.30 ± 4.41	1.04
		Balb/c 3T3	26.20 ± 2.71	
2.	Paroxetine (**2**)	PC-3	12.10 ± 0.37 *	1.23
		SH-SY5Y	18.10 ± 0.48 *	0.82
		Balb/c 3T3	14.90 ± 0.24	
3.	*N*-Ac-Paroxetine (**3**)	PC-3	45.30 ± 1.44 *	1.46
		SH-SY5Y	65.60 ± 1.01	1.01
		Balb/c 3T3	66.17 ± 2.35	
4.	Fluvoxamine (**4**)	PC-3	47.80 ± 2.85	1.09
		SH-SY5Y	89.40 ± 3.64 *	0.58
		Balb/c 3T3	52.00 ± 5.40	
5.	*N*-Ac-Fluvoxamine (**5**)	PC-3	92.23 ± 9.65	1.43
		SH-SY5Y	112.00 ± 3.85	1.21
		Balb/c 3T3	135.95 ± 3.95	
6.	KW117 (**6**)	PC-3	14.40 ± 1.70 *	2.65
		SH-SY5Y	25.40 ± 2.66	1.50
		Balb/c 3T3	38.10 ± 6.54	
7.	AZ05 (**7**)	PC-3	9.85 ± 0.79 *	2.48
		SH-SY5Y	17.30 ± 1.85 *	1.41
		Balb/c 3T3	24.40 ± 1.49	
8.	AZ07 (**8**)	PC-3	5.74 ± 1.02 *	2.00
		SH-SY5Y	9.15 ± 0.74	1.26
		Balb/c 3T3	11.50 ± 0.62	
9.	AB5A (**9**)	PC-3	27.20 ± 3.20 *	1.43
		SH-SY5Y	18.07 ± 1.68 *	2.15
		Balb/c 3T3	38.90 ± 4.08	
10.	AB9 (**10**)	PC-3	75.80 ± 6.46 *	0.29
		SH-SY5Y	39.33 ± 1.36	0.56
		Balb/c 3T3	22.20 ± 6.21	
11.	AB21 (**11**)	PC-3	18.00 ± 1.88	1.14
		SH-SY5Y	21.60 ± 1.07	0.95
		Balb/c 3T3	20.50 ± 3.50	
12.	AB22 (**12**)	PC-3	23.50 ± 2.21	1.51
		SH-SY5Y	35.00 ± 8.96	1.01
		Balb/c 3T3	35.50 ± 8.71	
13.	8-OH-DPAT (**13**)	PC-3	n.c.	n.c.
		SH-SY5Y	n.c.	n.c.
		Balb/c 3T3	n.c.	
14.	S14506 HCl (**14**)	PC-3	9.98 ± 1.26 *	2.06
		SH-SY5Y	4.27 ± 0.41	4.82
		Balb/c 3T3	20.60 ± 2.58	
15.	WAY100635 (**15**)	PC-3	43.30 ± 4.05 *	2.03
		SH-SY5Y	74.30 ± 11.80	1.18
		Balb/c 3T3	88.00 ± 13.53	

n.c.—IC_50_ not calculable; * *p* < 0.05 vs. Balb/c 3T3.

**Table 3:** Functional activity of new and classic ligands of 5-HT_1A_ receptor in HEK293 cells that overexpress the *5-HT1AR* gene.

Entry	Agonists	cAMP EC50 (M)	ERK1/2 EC50 (M)
1.	5-CT	8.20 × 10^−9^	11.50 × 10^−9^
2.	8-OH-DPAT	29.30 × 10^−9^	91.00 × 10^−9^
3.	S14506 HCl	25.40 × 10^−12^	62.02 × 10^−12^
	AB22	A	13.80 × 10^−9^
	Paroxetine	A	0.15 × 10^−6^
	*N*-Ac-Paroxetine	A	4.23 × 10^−6^
	**Antagonists**	**cAMP IC50 (M)**	**ERK1/2 IC50 (M)**
4.	WAY100635	1.40 × 10^−9^	8.23 × 10^−9^
5.	Spiperone	6.80 × 10^−6^	2.77 × 10^−6^
6.	Fluoxetine	0.54 × 10^−6^	0.83 × 10^−6^
7.	KW117	n.c.	7.18 × 10^−6^
8.	AB5A	96.90 × 10^−6^	17.90 × 10^−6^
9.	AB9	11.08 × 10^−6^	5.00 × 10^−6^
10.	AB21	2.00 × 10^−9^	0.78 × 10^−6^
11.	AB22	76.08 × 10^−6^	b
12.	AZ05	9.70 × 10^−6^	1.28 × 10^−6^
14.	AZ07	73.00 × 10^−8^	0.90 × 10^−6^
15.	Paroxetine	51.10 × 10^−9^	b
16.	*N*-Ac-Paroxetine	1.00 × 10^−6^	b
17.	Fluvoxamine	8.24 × 10^−6^	10.60 × 10^−6^
18.	*N*-Ac-Fluvoxamine	20.10 × 10^−6^	11.20 × 10^−6^

a—compound exhibits antagonistic activity on the cAMP pathway; b—compound exhibits; agonistic activity on the ERK1/2 pathway; n.c. —not calculable.

## Abbreviations

Akt	serine-threonine protein kinase B
pAkt1	phospho-Akt isoform 1
Balb/c 3T3	murine fibroblast cell line
cAMP	cyclic adenosine monophosphate
5-CT	5-carboxamidotryptamine
8-OH-DPAT	(R)-(+)-8- hydroxy-(2-*di*-*n*-propylamino)tetralin
DBU	1,8-diazabicyclo(5.4.0)undec-7-ene
DU-145	androgen-independent human prostate cancer cell line
EC_50_	concentration inducing 50% of maximal response
ECL	extracellular loop
EDTA	disodium ethylenediaminetetraacetic acid
ERK1/2	extracellular signal-regulated kinase 1 and 2
pERK1/2	phospho-ERK1/2
EGFP	enhanced green fluorescent protein
HEK	human embryonic kidney cell line
5-HT	5-hydroxytryptamine (serotonin)
5-HT_1A_ receptor	subtype of serotonin receptor 1A
IC_50_	concentration showing 50% inhibition of maximal response
LNCaP	androgen-sensitive human prostate adenocarcinoma
MAPK	mitogen activated protein kinase
MEK	acronym from MAPK/ERK kinase
MTT	(3-(4,5-dimethylthiazol-2-yl)-2,5-diphenyltetrazolium bromide)
NF-κB	nuclear transcription factor κB
PC-3	androgen-independent human prostate cancer cell line
PI3K	phosphatidylinositol-3-kinase
SEM	standard error of the mean
SERT	serotonin transporter
SH-SY5Y	human neuroblastoma-like cell line
SI	selectivity index
SSRI	selective serotonin reuptake inhibitor
TMH	transmembrane helices
VLS	virtual ligand screening
